# A novel antiviral strategy targeting human metapneumovirus through pH modulation in human airway epithelial cells

**DOI:** 10.1099/jgv.0.002274

**Published:** 2026-06-03

**Authors:** Ivana Alisandrea Daniels, Benjamin Gaston, Jessica Saunders, Laura Smith, Taiya Whitlow, Nastaha L. Tilston, Ryan F. Relich, Andrew Lunel, Michael D. Davis

**Affiliations:** 1 Department of Pharmacology and Toxicology, Indiana University School of Medicine, Indianapolis, Indiana, USA; 2 Herman B. Wells Center for Pediatric Research, Indiana University School of Medicine, Indianapolis, Indiana, USA; 3 Division of Pulmonology, Allergy and Sleep Medicine, Riley Hospital for Children, Indianapolis, Indiana, USA; 4 Department of Microbiology and Immunology, Indiana University School of Medicine, Indianapolis, Indiana, USA; 5 Department of Pathology and Laboratory Medicine, Indiana University School of Medicine, Indianapolis, Indiana, USA

**Keywords:** airway epithelial cells, airway pH, antiviral, fusion protein, human metapneumovirus, *Pneumoviridae*

## Abstract

Human metapneumovirus (hMPV) is a major cause of respiratory infections, particularly in vulnerable populations, yet no approved vaccines or targeted antivirals are available. pH-regulated processes, including airway epithelial physiology, endosomal acidification and viral fusion mediated by the fusion (F) protein, are critical for hMPV infection. This study evaluates PHOH-001, an inhaled alkaline buffer, as a potential therapeutic strategy to modulate airway pH and inhibit hMPV infection. Using rhMPV-GFP (green fluorescent protein-expressing hMPV), viral replication was assessed in primary human airway epithelial cells (HAECs). PHOH-001 significantly reduced GFP expression at 72 h post-infection in both submerged and air-liquid interface cultures, with effects comparable to those of the endosomal acidification inhibitor bafilomycin A1. In Vero E6 cells, PHOH-001 increased extracellular and intracellular pH in a dose-dependent manner and correspondingly reduced hMPV infection. In HAECs, PHOH-001 treatment reduced early virus–cell interactions, as measured by decreased cell-associated viral RNA under conditions restricting internalization. PHOH-001 also reduced viral replication, as measured by TCID_50_ assays and decreased syncytium formation. In parallel, PHOH-001 increased endosomal pH, consistent with altered endosomal acidification. Furthermore, PHOH-001 altered F protein localization and coincided with changes in actin organization, consistent with impaired viral spread. Collectively, these findings indicate that PHOH-001 modulates multiple pH-dependent steps in hMPV infection *in vitro* and support airway pH modulation as a potential antiviral strategy against hMPV.

## Introduction

Human metapneumovirus (hMPV) is a leading cause of acute respiratory tract infections worldwide. Like other members of the family *Pneumoviridae*, hMPV possesses a non-segmented, negative-sense, ssRNA genome, and viral particles are enveloped and adopt spherical morphologies [1,2]. Identified in 2001, hMPV has become recognized as a significant human respiratory pathogen. Disease manifestations can range from mild upper respiratory illness, including cough, nasal congestion and fever, to severe lower respiratory disease such as bronchiolitis and pneumonia [3,4]. Severe disease occurs most frequently among infants, older adults and immunocompromised individuals and is comparable in clinical severity to respiratory syncytial virus (RSV) infection [1,5, 6]. Nearly all children are infected by 5 years of age, and reinfections occur throughout life due to incomplete or short-lived immunity [7]. Recent studies have shown that paediatric lower respiratory tract infections, many caused by hMPV, lead to a twofold increased risk of adult death from respiratory disease [8]. Despite its high prevalence and significant contributions to long-term health outcomes, no hMPV-specific vaccines or therapies are approved for clinical use [9,11].

Like other pneumoviruses, hMPV infection begins with virion attachment to the respiratory epithelium, followed by fusion of the viral and host membranes, a process mediated exclusively by the viral fusion (F) protein [2]. Unlike RSV, hMPV entry is more heterogeneous and can occur either by direct fusion at the plasma membrane or following endocytosis, with fusion occurring at endosomal membranes to release the viral genome into the cytoplasm [12,15]. While direct fusion contributes to hMPV pathogenesis, multiple studies suggest that pH-dependent mechanisms, such as endocytosis and macropinocytosis, represent the predominant pathways for viral entry [13,16, 17]. These pathways are considered pH dependent, as endosomal maturation is accompanied by progressive acidification, which can promote conformational changes in the F protein that enable membrane fusion within intracellular compartments. Consequently, efficient endosomal trafficking and maturation are critical for successful genome release and downstream viral gene expression and replication. This requirement has implicated pH as an important regulator of F protein activation and has fuelled ongoing debate regarding the role of pH in hMPV entry.

Early biochemical and mutational analyses indicated that certain hMPV strains undergo robust low-pH-triggered conformational changes in the F protein, consistent with an endosomal fusion mechanism [18]. However, subsequent studies demonstrated that pH-triggered fusion occurs only in a subset of strains and that many isolates fuse efficiently at neutral pH [19,21]. To better understand the structural basis for this variability, analyses of prefusion-stabilized hMPV F reveal that the protein is stable at neutral pH and lacks the conserved pH-sensing residues typically required for acid-triggered fusion in other viral fusogens [22]. Together, these observations support the view that pH sensitivity is strain dependent and likely influenced by determinants within the F protein heptad repeats, cleavage loop and hydrophobic fusion peptide [20,22].

Despite these conflicting data regarding pH-triggered fusion, pH remains a biologically relevant antiviral target. hMPV primarily uses endocytic pathways such as clathrin-mediated endocytosis and macropinocytosis to access acidified endosomes, suggesting that endosomal pH may modulate entry efficiency [13,23]. Additionally, host airway surface liquid (ASL) pH regulates epithelial defence mechanisms, protease activity and viral stability, and perturbations in ASL pH can influence infection dynamics [24,29]. Lastly, the F protein conformational landscape remains sensitive to environmental factors, including ionic strength and protonation of specific residues, which may shift the balance between the prefusion and post-fusion states [22,30,32]. Together, these observations suggest that therapeutic modulation of pH, either by disrupting endosomal maturation or by altering the extracellular airway environment, may interfere with critical steps in viral entry. However, whether modulation of airway epithelial pH can be therapeutically leveraged to disrupt hMPV infection and F protein-mediated viral spread in primary airway epithelium remains unclear.

Based on this rationale, we hypothesized that pharmacological elevation of airway epithelial cell pH would impair hMPV infection by disrupting pH-sensitive entry and post-entry processes. To test this hypothesis, we developed PHOH-001 (formerly ‘Optate’, Atelerix Life Sciences, Indianapolis, IN), an inhaled alkaline glycine buffer that has been shown to safely raise human airway pH *in vivo* [33,34]. In primary human airway epithelial cells (HAECs) *in vitro*, PHOH-001 raises intracellular pH and has been associated with altered expression of genes involved in endosomal trafficking, including *Rab5*, *Rab*7, *Rab9* and *Rab11* [33]. PHOH-001 also exhibits broad antiviral activity, reducing severe acute respiratory syndrome coronavirus 2 (SARS-CoV-2) and RSV infection in HAECs [33,35]. Here, we examine the effects of PHOH-001 on hMPV entry, replication and F protein-mediated membrane fusion in organotypic human airway epithelial cultures to assess the potential of pH modulation as a therapeutic strategy for hMPV infection.

## Methods

### Cells and virus

Primary HAECs from the lower respiratory tract (bronchial) were obtained from donors at Indiana University School of Medicine. Paediatric primary HAECs were purchased from Lifeline Cell Technology (#07783) (Table S1, available in the online Supplementary Material). Cells were cultured under submerged or air–liquid interface (ALI) conditions for 7 or 21 days, respectively, as previously described [33]. Experiments with HAECs were conducted at passages 2–6. Vero E6 cells (African green monkey kidney cells) were maintained in Dulbecco’s Modified Eagle Medium (DMEM; Gibco, #11965092) supplemented with 10% non-heat-inactivated FBS (Cytiva, SH3008003) and 1% penicillin/streptomycin (Gibco, #15140122) at passage 4. A green fluorescent protein-expressing recombinant human metapneumovirus [rhMPV-GFP; ViraTree, MPV-GFP1, #M121, CAN97-83 (A2)] was used at virus passages 4–5. Viral stocks were propagated in LLC-MK2 cells (ATCC CCL-7) and stored in suspension medium consisting of Opti-MEM, sucrose and l-glutamic acid [36]. Viral titres were ≥7.0 log_10_ TCID_50_ ml^−1^.

All experiments were carried out in humidified conditions at 37 °C under 5% CO_2_.

### Viral growth kinetics

Vero E6 cells and HAECs were seeded in black-walled, clear-bottom 96-well plates (Corning, #3603) and grown to confluency. Vero E6 and HAEC monolayers were rinsed with PBS (Gibco, #10010023) and infected with rhMPV-GFP at an m.o.i. of 0.01, 0.1 or 1 (*n*=3). The virus was diluted in FluoroBrite™ DMEM (Gibco, A1896701), a phenol red-free medium, to minimize autofluorescence during live cell imaging. GFP signals were quantified at specified timepoints using a FLUOstar Omega plate reader (BMG Labtech), and viral growth kinetics were plotted as relative light units (RLU). Supernatants collected at each timepoint were titrated using TCID_50_ assays to determine infectious viral titres.

### TCID_50_ assay

Vero E6 cells were seeded at 1.2×10^4^ cells per well in 96-well plates (Corning, #3360). After 24 h, monolayers were inoculated with tenfold serial dilutions of infected supernatant. At 5–7 days post-infection, wells were scored for the presence of GFP-positive infection. Viral titres were calculated and expressed as TCID_50_ ml^−1^. The limit of detection (LOD) for the TCID_50_ assay was calculated based on the lowest dilution tested (10^−1^) and an inoculation volume of 200 µl per well, resulting in an LOD of 1.7 log_10_ TCID_50_ ml^−1^.

### Viral attachment assay

To distinguish effects on viral attachment from downstream steps in the viral life cycle, HAEC monolayers cultured under submerged conditions were pre-chilled at 4 °C for 30 min to inhibit endocytosis and membrane fusion while allowing viral binding. rhMPV-GFP was diluted in Opti-MEM (Gibco, #31985070) and added to cells (m.o.i.=1) in the presence of PHOH-001 or PBS control (1:1 dilution). Plates were incubated at 4 °C for 1 h to permit viral attachment, with gentle rocking performed every 15 min to ensure even distribution of the inoculum.

Following incubation, the inoculum was removed, and cells were washed three times with ice-cold PBS to remove unbound virus. Cells were immediately lysed, and total RNA was extracted. Viral RNA levels were quantified by reverse transcription quantitative PCR (RT-qPCR), as described below, to evaluate the relative amount of cell-associated viral RNA under attachment conditions. Experiments were performed twice, each using one biological replicate with three technical replicates per condition.

### Reverse transcription quantitative PCR

At 72 h post-infection (hpi), HAECs from infected submerged models treated with PHOH-001 or PBS were lysed, and total RNA was extracted using a RNeasy® Plus Mini Kit (Qiagen, #74134) following the manufacturer’s instructions. RNA samples were reverse transcribed to cDNA using the Maxima H Minus cDNA Synthesis Kit (Thermo Fisher Scientific, M1681) according to the manufacturer’s protocol. cDNA was eluted in 50 µl of Ambion Nuclease-Free Water (Invitrogen), quantified using a Biospectrometer (Eppendorf) and diluted to a final concentration of 100 ng per sample.

Primers, probe and a synthetic gene block were designed based on the hMPV F protein gene sequence (NCBI accession: AY145296, prototype A2 subtype CAN97-83). Primer and probe sequences were checked for cross-reactivity, self-dimerization, hairpin formation and secondary structure using NCBI blast, OligoCalc and OligoAnalyzer. Complete sequences are provided in Table S2; primers and gene block were synthesized by Integrated DNA Technologies (IDT), and the probe was purchased from Applied Biosystems.

RT-qPCR was performed using a QuantStudio 3 instrument (Applied Biosystems, A28567) with TaqMan Fast Virus 1-Step Multiplex Master Mix (Applied Biosystems, 5555532). Absolute quantification was determined using a standard curve generated using tenfold serial dilutions of the gene block, each tested in triplicate [37]. Each 25 µl PCR reaction contained 400 nM forward primer, 400 nM reverse primer, 250 nM TaqMan probe (FAM), 2 µl of cDNA and TaqMan Fast Virus 1-Step MM. Thermal cycling was performed using a fast run protocol: 5 min at 50 °C for reverse transcription, followed by 20 s at 95 °C for initial denaturation and then 40 cycles of 3 s at 95 °C for denaturation and 30 s at 60 °C for annealing.

### GFP-based quantification of infection via fluorescence microscopy

Confluent HAEC monolayers in 12-well plates (Corning, #3513) were infected with rhMPV-GFP (m.o.i.=1). PHOH-001 was prepared by a compounding pharmacy at 120 mM and verified for purity, potency, osmolality (~330 mOsm), pH (9.8) and sterility (IND #139144; Arena District Pharmacy) [34]. PHOH-001 (120 mM) or control (PBS; pH 7.2) was co-administered with rhMPV-GFP diluted in Opti-MEM (m.o.i.=1) (1 : 1 dilution) for 4 h; bafilomycin (100 nM; Millipore Sigma, #508409) served as an additional treatment group in ALI experiments and was co-administered with rhMPV-GFP for 4 h. Supernatants were collected at 4 hpi and every 24 h for 72 h for both submerged and ALI cultures. Submerged cultures were treated daily during medium changes; ALI cultures were treated apically for 20 min twice daily with daily basolateral medium changes supplemented with 0.3 µg ml^−1^ L-(tosylamido-2-phenyl) ethyl chloromethyl ketone (TPCK)-treated trypsin (Thermo Scientific, #20233).

Images were acquired every 24 h for 3 days using an EVOS M5000 microscope (Thermo Fisher Scientific) with an EVOS™ Light Cube, GFP 2.0 (Thermo Fisher Scientific, AMEP4951) at 4× magnification (*n*=6). GFP-positive regions were segmented in Fiji [38] using noise-reduction parameters and thresholding, followed by particle analysis. The GFP-positive area was quantified and compared across treatment groups to assess infection at 72 hpi.

### Automated Western blot

At 72 hpi, HAECs from infected and treated submerged and ALI cultures were lysed in 50 µl RIPA buffer (Sigma-Aldrich, R0278) supplemented with 1% protease inhibitor (Millipore, #539134). Lysates were centrifuged at 14,000 ***g*** for 10 min at 4 °C, and supernatants were stored at −80 °C. Protein concentration was measured using the Pierce BCA Protein Assay Kit (Thermo Fisher Scientific, #23250). Samples were diluted in 1X Sample Buffer to 0.25 µg µl^−1^ and analysed using the Jess automated western capillary system (ProteinSimple, #004-650). GFP was detected using a rabbit monoclonal antibody (1:50; Cell Signaling Technology; 2956) normalized to *β*-actin detected with a mouse monoclonal antibody (1:25; Cell Signaling Technology; 3700). hMPV F protein was detected using a rabbit polyclonal antibody (1 : 25; AntibodySystem, PVV22901) normalized to *β*-actin (1 : 25; Cell Signaling Technology; 3700). Chemiluminescent signals were quantified using Compass for SW software (ProteinSimple).

### GFP ELISA

HAECs from infected and treated submerged and ALI cultures were lysed at 72 hpi in 50 µl 1 X Cell Extraction Buffer PTR (CatchPoint® SimpleStep GFP ELISA; Abcam, ab229403). Protein concentrations were measured (Pierce BCA Protein Assay Kit; Thermo Fisher Scientific), and lysates were centrifuged at 14,000 ***g*** for 10 min at 4 °C and stored at −80 °C. Prior to analysis, samples were diluted to the required concentration in 1 X Cell Extraction Buffer PTR, and a standard curve was generated using recombinant GFP provided with the kit. HRP fluorescence was quantified using a FLUOstar Omega plate reader following the manufacturer’s protocol, and GFP concentrations were calculated using a four-parameter logistic regression model.

### Dose-response assays

Vero E6 cells were seeded at 1.2×10⁴ cells per well in 96-well plates. Upon confluency, cells were infected with rhMPV-GFP (m.o.i.=0.1) and immediately treated with PHOH-001 at 120 mM, followed by 1:2 serial dilutions across all 12 columns (*n*=8). Plates were incubated with PHOH-001 for 48 h. Fluorescence was measured using a FLUOstar Omega plate reader, and whole-plate images were acquired using an Odyssey M Imager (LI-COR). To ensure accurate interpretation of the results, the pH of the PHOH-001 stock and its serial dilutions were assessed simultaneously with the experiment using a pH meter (Mettler-Toledo).

### Intracellular pH assay

Vero E6 cells were seeded in black-walled, clear-bottom 96-well plates. Once confluent, cells were washed with Live Cell Imaging Solution (Thermo Fisher Scientific, A59688DJ) and stained with pHrodo™ Red Intracellular pH Dye according to the manufacturer’s instructions (Thermo Fisher Scientific, P36600). Cells were treated with PHOH-001 at 120 mM followed by 1:2 serial dilutions in FluoroBrite™ DMEM across 12 columns (*n*=8). pHrodo Red fluorescence was quantified using a FLUOstar Omega plate reader (excitation 544 nm, emission 590 nm) at 30 min post-treatment. Immediately following fluorescence measurements, plates were imaged using an Odyssey M full-plate imager to ensure consistency between quantitative and imaging-based readouts. Higher-resolution fluorescence images were subsequently acquired using an EVOS M5000 microscope (Thermo Fisher Scientific) equipped with an EVOS™ Light Cube, RFP 2.0 (Thermo Fisher Scientific, AMEP4952) at 4× magnification.

### Endosomal pH analysis

Endosomal pH was assessed using pHrodo™ Red dextran (Thermo Fisher Scientific, P10361), a pH-sensitive fluorescent dextran that accumulates within endocytic vesicles. HAECs were incubated with pHrodo Red dextran according to the manufacturer’s instructions to allow endosomal uptake. Cells were counterstained with Hoechst 33342, trihydrochloride and trihydrate (Thermo Fisher Scientific, H1399) according to the manufacturer’s protocol to facilitate identification of cell nuclei during imaging. Following loading and washing, cells were treated with PHOH-001 or control conditions as indicated. Fluorescence images were acquired at 60× magnification using an EVOS M5000 microscope. Fluorescence intensity was quantified using Fiji by measuring integrated density within regions of interest corresponding to individual cells. Increased fluorescence intensity corresponds to increased endosomal acidification, whereas decreased fluorescence is consistent with reduced endosomal acidification.

### Syncytium assay

Confluent HAEC monolayers in black-walled, clear-bottom 96-well plates were co-inoculated with rhMPV-GFP (m.o.i.=1) and PHOH-001 or PBS (1:1 dilution) for 4 h. At 4 hpi, the inoculum was removed, the monolayers were rinsed with PBS, and fresh medium containing PHOH-001 or PBS (1 : 1 dilution) was added. Cultures were maintained for 72 h with daily medium replacement containing the corresponding treatment.

At 72 hpi, monolayers were fixed with 4% paraformaldehyde (PFA) for 15 min and permeabilized with PBS containing 0.1% Triton X-100 (Sigma-Aldrich, #9036-19-5) for 20 min. GFP signal remained stable after fixation and did not require additional staining. Upon fixation, the cell monolayers were blocked for 1 h using 5% donkey serum (Rockland, D220-00-0050). Cell membranes were then stained with a monoclonal antibody against E-cadherin (1:200; BD Biosciences, #610181), and nuclei were stained with Hoechst 33342, trihydrochloride and trihydrate following the manufacturer’s instructions. After washing, samples were incubated with goat anti-mouse IgG 647 (Invitrogen, A32728) followed by a final wash step. Immunofluorescence imaging was acquired at 60× magnification using an EVOS M5000 microscope (Thermo Fisher Scientific) (*n*=25).

Syncytia were quantified using CellProfiler (v4.2.8) [39] with a custom pipeline for segmenting nuclei, GFP-positive regions and E-cadherin-defined cell borders. Nuclei within GFP-positive, membrane-bounded regions were counted, and cells containing ≥2 nuclei were classified as syncytia. The frequency of syncytia was calculated by dividing the number of nuclei within syncytia by the total number of nuclei per field and multiplying by 100, providing the percent of cells participating in syncytia for each treatment group. Total nuclei were used rather than total GFP-positive cells because imaging at 60× provided a limited field of view, and using total nuclei gave a consistent measure across images.

### Fusion protein analysis via confocal microscopy

HAECs were seeded on glass coverslips at 5.0×10^4^ cells per coverslip. Upon reaching confluency, cells were co-inoculated with rhMPV-GFP (m.o.i.=1) and PHOH-001 or PBS (120 mM) (1:1 dilution) for 4 h. At 4 hpi, the inoculum was removed, the monolayers were rinsed with PBS and fresh medium containing the corresponding treatment was added. Cultures were maintained for 72 h with daily medium and treatment replacement.

At 72 hpi, coverslips were fixed with 4% PFA for 15 min and permeabilized with PBS containing 0.1% Triton X-100 for 20 min. GFP signal remained stable after fixation and required no additional staining. Coverslips were blocked with 5% donkey serum for 1 h and then incubated with a monoclonal antibody against hMPV F protein (1 : 300; Abcam, ab94800) and phalloidin (Thermo Fisher Scientific, A22283) to label filamentous actin for cell segmentation. After washing, samples were incubated with goat anti-mouse IgG Alexa Fluor 647, followed by final washes and mounting with ProLong™ Gold antifade reagent containing DAPI (Invitrogen, P36931).

Images were acquired on a Zeiss LSM 800 AxioObserver with Airyscan using a 20× and 63× Plan-Apochromat objective (*n*=25 cells per condition). Image analysis for 63× images was performed using CellProfiler. Phalloidin staining was used to delineate individual cell boundaries and enable segmentation of single cells, while GFP signal was used to identify infected cells. A plasma membrane-proximal peripheral region was generated by uniformly eroding the phalloidin-defined whole-cell mask inward by a fixed distance of 5 µm, and the cytoplasmic region was defined as the remaining intracellular area. Integrated fluorescence intensity of F protein was measured within the whole-cell and peripheral regions by summing pixel intensity values within each region of interest. Peripheral enrichment was calculated on a per-cell basis as the ratio of integrated F protein intensity within the peripheral region to the integrated intensity within the whole cell and is reported as a percentage. Values were compared across treatment conditions.

### Lactate dehydrogenase assay

Cytotoxicity following PHOH-001 treatment was assessed by measuring lactate dehydrogenase (LDH) release from airway epithelial cells. Cells were treated with increasing concentrations of PHOH-001 (0–120 mM) and incubated at 37 °C for 4, 12 or 24 h. At each time point, cell culture supernatants were collected, and LDH release was quantified using a colorimetric LDH assay according to the manufacturer’s instructions (LDH Cytotoxicity Assay Kit, Cayman Chemical, Cat. No. 601170). Cytotoxicity was calculated relative to spontaneous LDH release (untreated cells) and maximum LDH release (lysis control) using the standard formula: (sample − spontaneous release) / (maximum release − spontaneous release) × 100. All conditions were assessed in technical triplicate, and values represent the mean of three replicates per condition. Values below zero following normalization were set to zero for visualization.

### Data analysis

All statistical analyses were performed using GraphPad Prism (version 10.6.1). For comparisons between two groups, a two-sample, two-tailed t-test was applied to data with a Gaussian distribution, whereas the Wilcoxon rank-sum test was used for non-Gaussian-distributed data. For experiments involving more than two groups, robust one-way ANOVA models were applied, followed by pairwise comparisons using Tukey’s post hoc test. For ELISA assays, a four-parameter logistic regression model was used according to the manufacturer’s instructions. Schematics were created using BioRender.com. Biological replicates represent independent donor-derived HAEC cultures, whereas technical replicates represent independent wells or fields within the same culture. To differentiate donor origins, biological replicates are colour-coded accordingly. For all data displayed, circles represent PBS-treated controls, squares represent PHOH-001-treated samples, diamonds represent bafilomycin-treated samples and triangles represent uninfected controls. Statistical significance was defined as a *P*-value ≤0.05.

### Imaging quantification codes

The CellProfiler pipelines and Fiji scripts used for imaging-based quantification of syncytium formation and F protein localization are available from the corresponding author upon request (A.L.).

## Results

### Recombinant hMPV-GFP exhibits robust replication kinetics and validates GFP as a quantitative reporter in primary HAECs

**Fig. 1.**
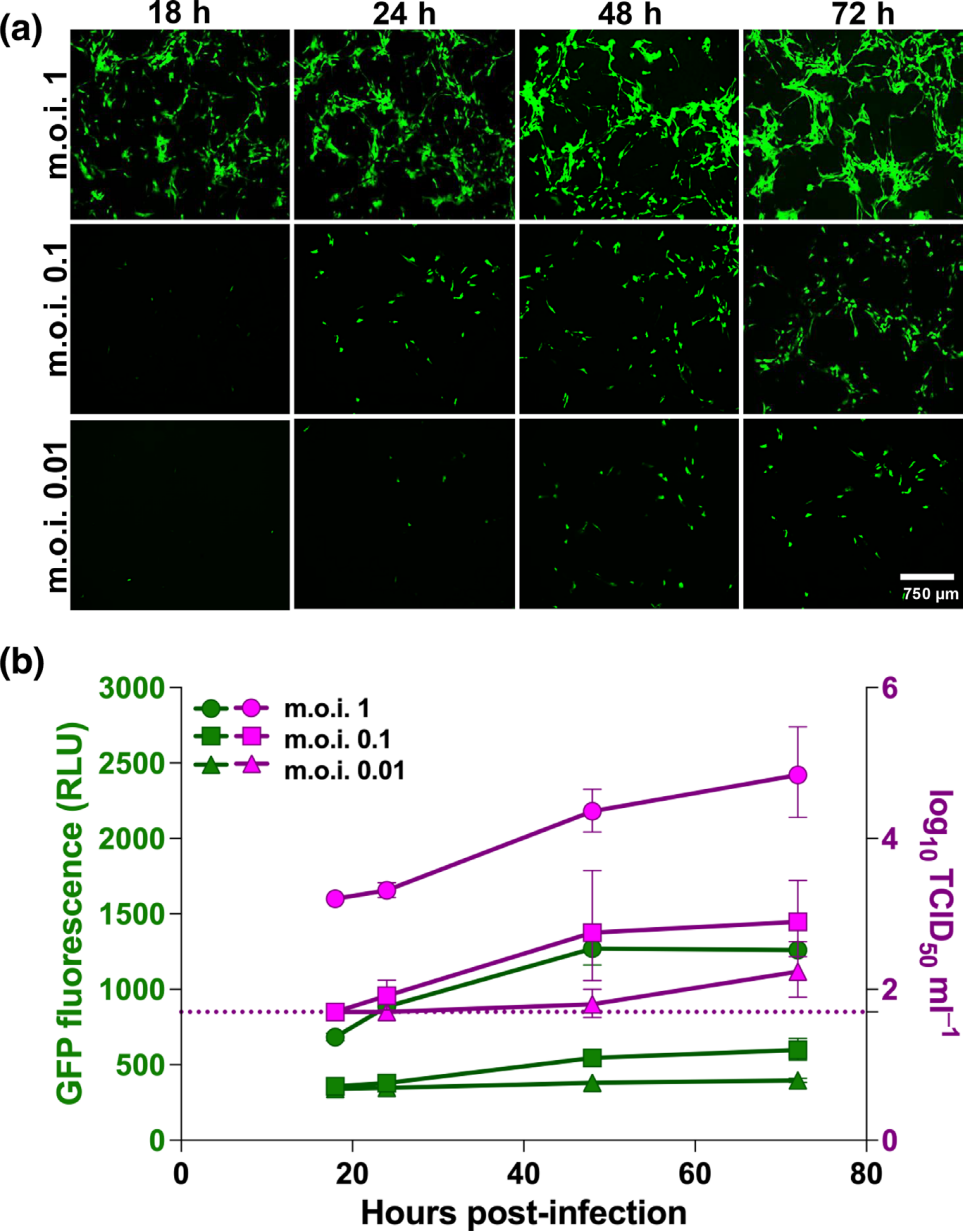
Validation of GFP fluorescence as a quantitative reporter of hMPV infection in primary HAECs. (**a**) Representative fluorescence images of HAECs infected with rhMPV-GFP at m.o.i. of 0.01, 0.1 or 1 at 18, 24, 48 and 72 hpi. GFP-positive signal increases over time in an m.o.i.-dependent manner. Images were acquired at 4× magnification. Scale bar=750 µm. (**b**) Quantification of GFP fluorescence (RLU; left y-axis) and infectious titres (TCID_50_ ml^−1^; right y-axis) over time. RLU and TCID_50_ increased in parallel in an m.o.i.- and time-dependent manner. Dashed line indicates the TCID_50_ assay LOD (1.7 log10 TCID_50_ ml^−1^), with values below the limit plotted at the threshold. Data represent mean±sd from three technical replicates.

To establish a rapid and quantitative approach for monitoring hMPV infection, we assessed the replication dynamics of a recombinant GFP-expressing hMPV [rhMPV-GFP; CAN97-83 (A2)] in Vero E6 cells and primary HAECs. Assay parameters and infection conditions were first optimized in Vero E6 cells prior to evaluation in HAECs (Fig. S1). HAEC cultures were then inoculated at m.o.i. of 0.01, 0.1 and 1. GFP fluorescence increased in a clear m.o.i.- and time-dependent manner, with GFP-positive signal expanding across the epithelial surface over the course of infection (Fig. 1a). Quantification of infection by both RLU and infectious titres (TCID_50_) demonstrated that GFP fluorescence closely paralleled viral replication kinetics across all inoculation parameters tested (Fig. 1b). RLU values progressively increased at each time point and strongly correlated with corresponding increases in infectious titres, confirming that GFP fluorescence provides a reliable and quantitative surrogate for productive hMPV replication in primary HAECs. These findings validate the rhMPV-GFP infection model as an effective tool for downstream mechanistic and antiviral studies in the airway epithelium.

### PHOH-001 significantly reduces hMPV infection in primary HAECs under submerged culture

**Fig. 2.**
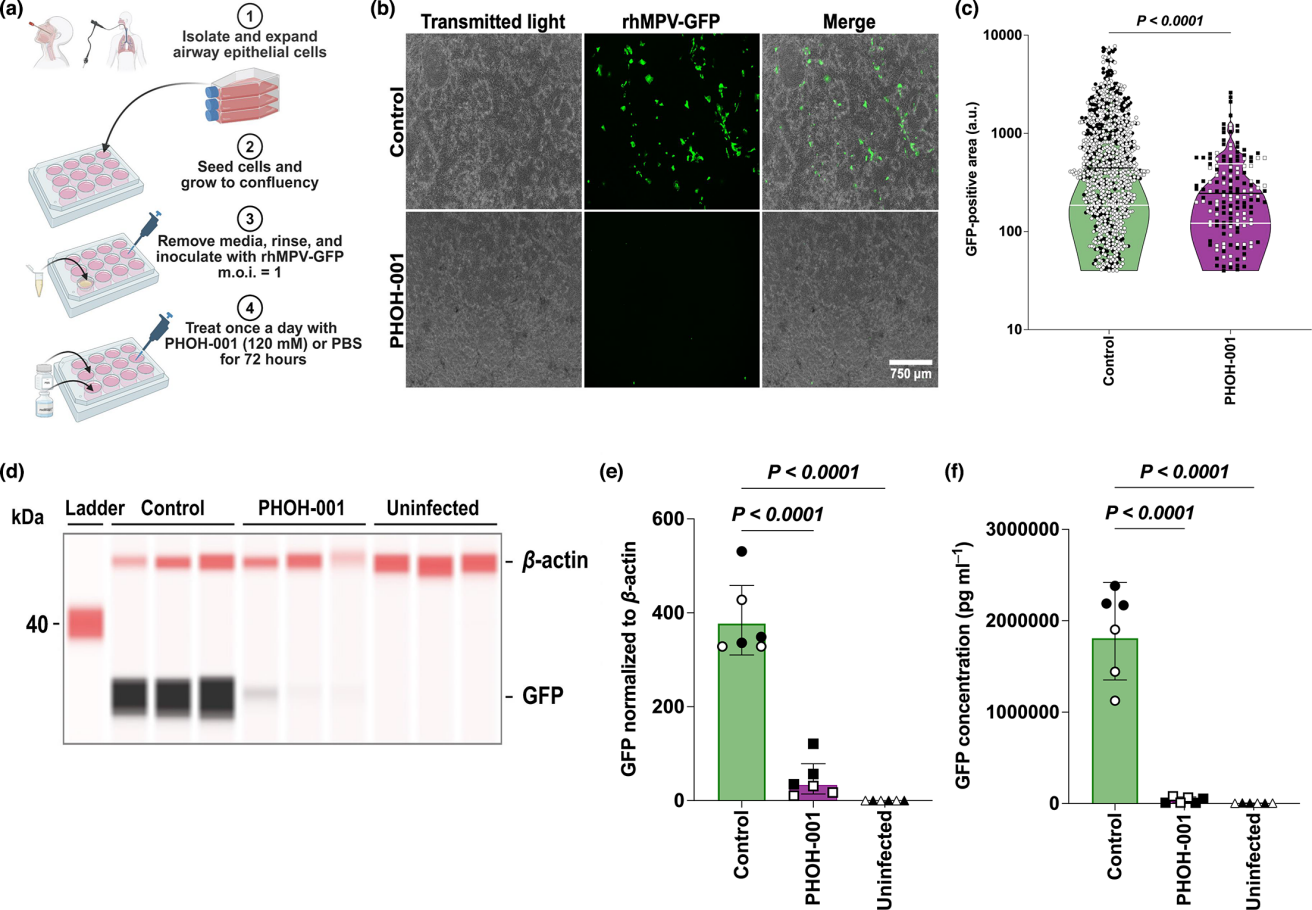
PHOH-001 reduces hMPV infection in submerged HAECs. (**a**) Experimental design. Primary HAECs were infected with rhMPV-GFP (m.o.i.=1) and treated daily with PHOH-001 or PBS for 72 h. (**b**) Representative fluorescence images at 72 hpi show reduced GFP signal in PHOH-001-treated cells relative to PBS controls. Images were acquired at 4× magnification. Scale bar=750 µm. (**c**) Violin plot quantification of GFP-positive areas demonstrates that GFP signal was significantly reduced in PHOH-001-treated cells compared to PBS. Violin plots were visually truncated to enhance readability; all individual data points are shown, with colours indicating biological replicates; black line, median; white lines, upper and lower quartiles. Data represent individual data points with mean±sd from two biological replicates, each with six technical replicates (*P*<0.05, two-tailed unpaired Student’s t-test). (**d**) Western blot analysis of GFP expression at 72 hpi. (**e**) Quantification of GFP normalized to *β*-actin confirms reduced GFP levels in PHOH-001-treated cells. Data points are colour-coded by biological replicate. Data represent mean±sd from two biological replicates, each with three technical replicates (*P*<0.05, one-way ANOVA). (**f**) ELISA quantification of GFP signal further confirms reduced GFP levels in PHOH-001-treated cells. Data points are colour-coded by biological replicate. Data represent mean±sd from two biological replicates, each with three technical replicates (*P*<0.05, one-way ANOVA).

To evaluate whether PHOH-001, an alkaline buffer, inhibits hMPV infection via pH modulation, primary HAECs were co-inoculated with rhMPV-GFP (m.o.i.=1) and either PHOH-001 or PBS control buffer. Cells were treated daily with PHOH-001 or PBS post-inoculation (Fig. 2a). Fluorescence microscopy at 72 hpi revealed reduced GFP fluorescence in PHOH-001-treated cells compared to controls (Fig. 2b). Quantification using Fiji software confirmed a significant decrease in GFP-positive areas in PHOH-001-treated HAECs (Fig. 2c). Western blot analysis confirmed decreased GFP expression in PHOH-001-treated cells (Fig. 2d), and quantification of GFP normalized to *β*-actin confirmed a consistent reduction (Fig. 2e). Additionally, ELISA assays were performed as a secondary protein readout, further supporting the Western blot data by demonstrating significantly reduced GFP levels in PHOH-001-treated cells (Fig. 2f). Together, these results indicate that PHOH-001 reduces hMPV infection in primary HAECs *in vitro*.

### PHOH-001 significantly reduces hMPV infection in organotypic, differentiated primary HAECs

**Fig. 3.**
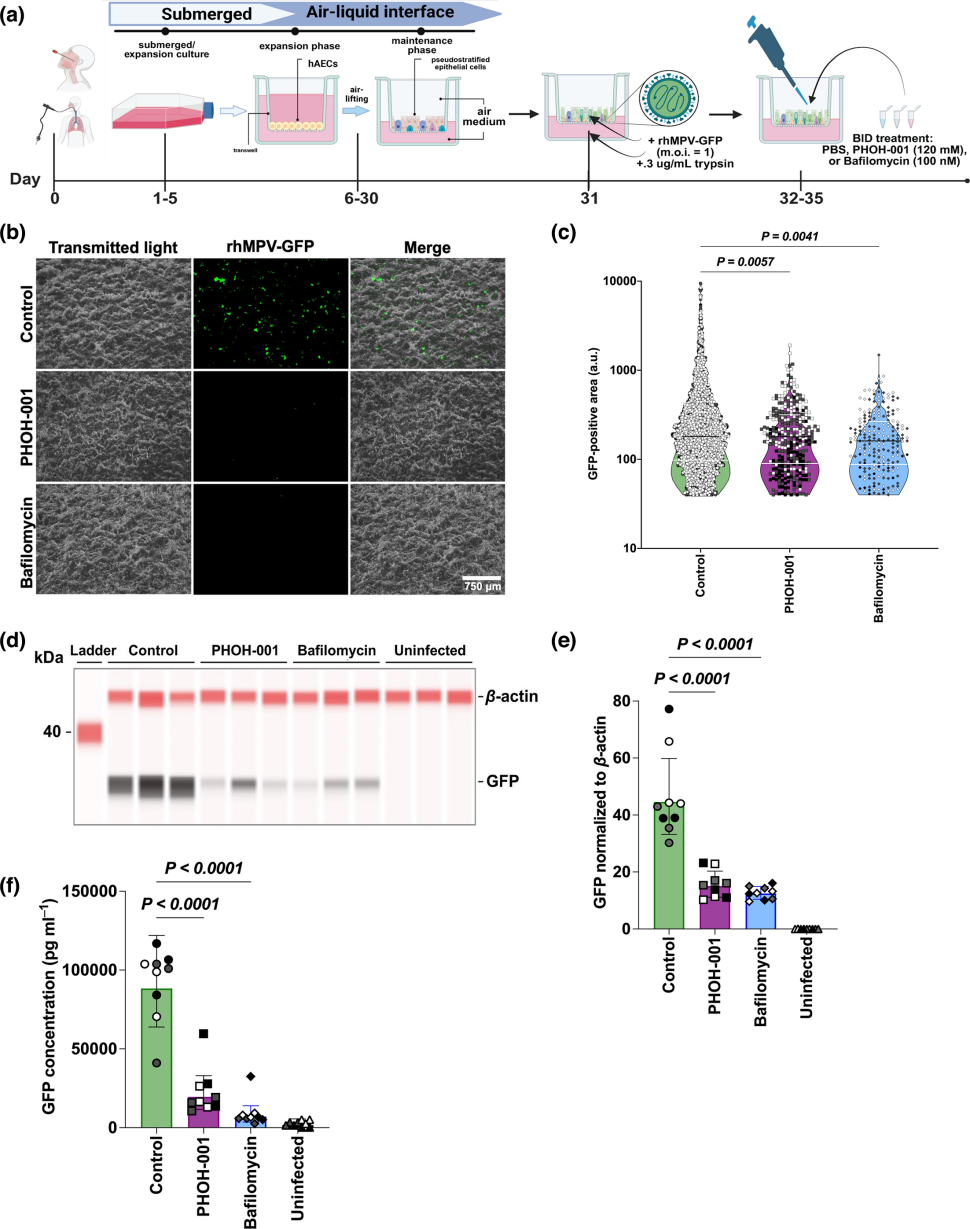
PHOH-001 reduces hMPV infection in HAECs cultured at ALI. (**a**) Experimental design; differentiated HAECs were inoculated with rhMPV-GFP (m.o.i.=1) and treated apically with PHOH-001, bafilomycin or PBS. Cells were treated twice daily, with once-daily basolateral media changes supplemented with TPCK-trypsin. (**b**) Representative fluorescence images at 72 hpi show reduced GFP signal in PHOH-001-treated cells relative to PBS controls. Images were acquired at 4× magnification. Scale bar=750 µm. (**c**) Violin plot quantification of GFP-positive areas demonstrates a significant reduction in PHOH-001-treated cells compared to PBS, with no significant difference relative to bafilomycin. Violin plots were visually truncated to enhance readability; all individual data points are shown, with colours indicating biological replicates; black line, median; white lines, upper and lower quartiles. Data represent individual data points with mean±sd from three biological replicates, each with six technical replicates (*P*<0.05, one-way ANOVA). (**d**) Western blot analysis of GFP expression at 72 hpi. (**e**) Quantification of GFP normalized to *β*-actin confirms reduced GFP expression in PHOH-001-treated cells relative to PBS, with no significant difference relative to bafilomycin. Data points are colour-coded by biological replicate. Data represent mean±sd from three biological replicates, each with three technical replicates (*P*<0.05, one-way ANOVA). (**f**) ELISA analysis corroborates reduced GFP expression in PHOH-001-treated cells. Data points are colour-coded by biological replicate. Data represent mean±sd from three biological replicates, each with three technical replicates (*P*<0.05, one-way ANOVA).

To assess whether PHOH-001 inhibits hMPV infection through pH modulation, primary HAECs were cultured under ALI conditions, generating a differentiated, pseudostratified, ciliated epithelium that mimics the *in vivo* airway epithelium. Bafilomycin A1, a well-established inhibitor of endosomal acidification and a known inhibitor of hMPV entry, was included as a positive control [13,19]. HAECs were co-inoculated with rhMPV-GFP (m.o.i.=1) and PHOH-001, bafilomycin or PBS control buffer on the apical side of the membrane (Fig. 3a). TPCK-treated trypsin was added to the basolateral media to cleave the F0 precursor and promote viral replication [15,20, 36]. Fluorescence microscopy at 72 hpi revealed a reduction in GFP signal in PHOH-001-treated cells compared to PBS-treated controls (Fig. 3b). Quantification of GFP fluorescence using Fiji showed a significant decrease in GFP signal in PHOH-001-treated HAECs relative to PBS-treated controls (Fig. 3c), with no significant difference observed between PHOH-001 and bafilomycin treatments. Western blot analysis also showed reduced GFP expression in PHOH-001-treated cells (Fig. 3d), with quantification showing a significant decrease in GFP normalized to *β*-actin (Fig. 3e). Additionally, ELISA analysis of GFP expression further supported the inhibitory effect of PHOH-001, showing a significant decrease in GFP levels in PHOH-001-treated cells compared to PBS controls (Fig. 3f). Notably, no significant differences in GFP expression were found between PHOH-001 and bafilomycin-treated HAECs. Taken together, these data demonstrate reduced hMPV infection in PHOH-001-treated HAECs cultured at ALI, supporting its potential for further investigation.

### PHOH-001 reduces hMPV infection in a dose-dependent manner associated with pH modulation

**Fig. 4.**
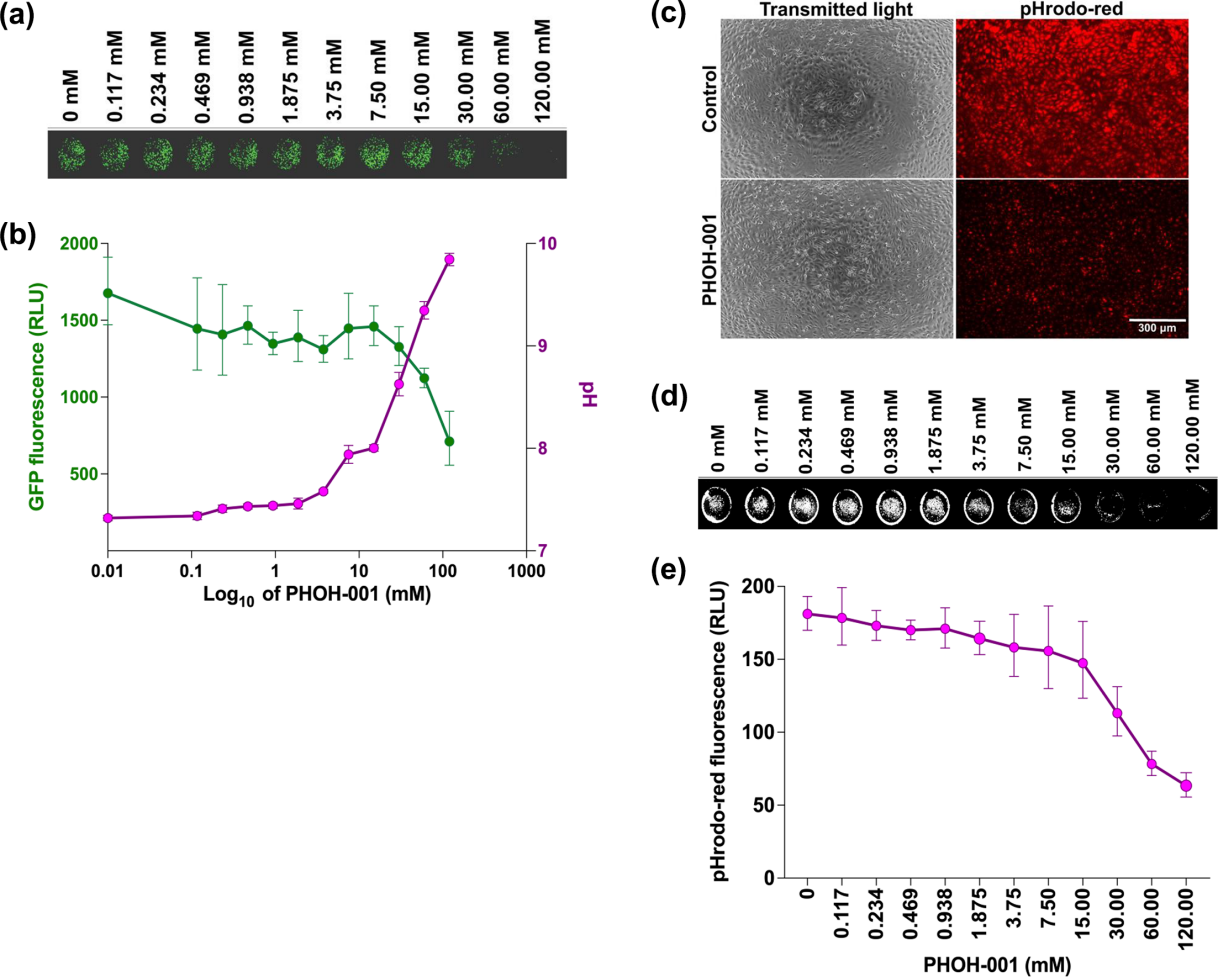
Dose-dependent effects of PHOH-001 on hMPV infection and pH. (**a**) Vero E6 cells were infected with rhMPV-GFP (m.o.i.=0.1) and treated with increasing concentrations of PHOH-001. Whole-plate fluorescence images were acquired, showing a dose-dependent reduction in GFP signal. The image shown corresponds to a single row of the 96-well plate. (**b**) Quantification of GFP signal (RLU; left y-axis) plotted alongside the pH of PHOH-001 buffer (pH; right y-axis). GFP signal decreased as PHOH-001 concentration increased, with a corresponding increase in pH. Data represent mean±sd from eight technical replicates for RLU and three technical replicates for pH. (**c**) Representative fluorescence images of pHrodo Red intracellular pH assays in Vero E6 cells treated with 0 mM or 120 mM PHOH-001. Images were acquired at 10× magnification. Scale bar=300 µm. (**d**) Representative whole-plate image showing pHrodo Red fluorescence in Vero E6 cells treated with PHOH-001 (120 mM) followed by 1 : 2 serial dilutions across a 96-well plate. Images were acquired with an Odyssey M Imager immediately following fluorescence quantification by a plate reader to confirm consistency between fluorescence intensity and spatial signal distribution. The image shown corresponds to a single row of the 96-well plate from this experiment. (**e**) Quantification of pHrodo Red fluorescence in Vero E6 cells treated with increasing concentrations of PHOH-001. Fluorescence was measured by a plate reader and expressed as RLU. Because pHrodo Red fluorescence increases with decreasing intracellular pH, the observed dose-dependent decrease in fluorescence indicates relative intracellular alkalinization with higher PHOH-001 concentrations. Data represent mean±sd from eight technical replicates.

To investigate the antiviral effects of PHOH-001 against hMPV, Vero E6 cells were infected with rhMPV-GFP (m.o.i.=0.1) and treated with increasing concentrations of PHOH-001. Fluorescence imaging at 48 hpi revealed a dose-dependent reduction in GFP signal (Fig. 4a). Quantification of GFP fluorescence (RLU) and comparison to the corresponding PHOH-001 buffer pH showed that as GFP signal decreased, PHOH-001 pH increased, consistent with an association between reduced infection and pH modulation (Fig. 4b). Intracellular pH was assayed using the pH-sensitive dye pHrodo Red, which increases in fluorescence as pH decreases, enabling visualization of intracellular acidification (Fig. 4c). Fluorescence measurements indicated that intracellular pH increased with higher PHOH-001 concentrations, as reflected by reduced pHrodo Red signal (Fig. 4d, e). Western blot and RT-qPCR analysis across a narrower dose range further confirmed this trend, showing reduced GFP protein and hMPV F gene expression with increasing PHOH-001 concentrations (Fig. S2). Together, these results demonstrate a dose-dependent reduction in hMPV infection associated with increases in extracellular and intracellular pH.

### PHOH-001 is associated with altered endosomal pH in primary HAECs

**Fig. 5.**
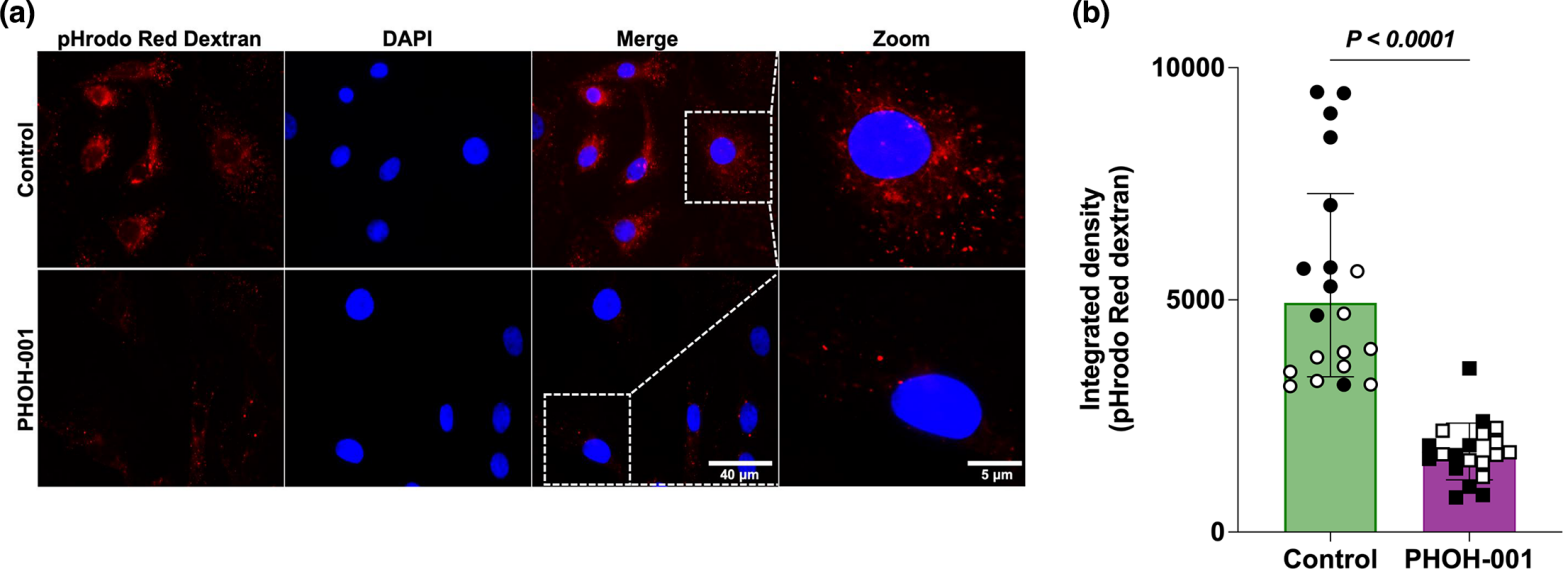
PHOH-001 alters endosomal acidification in primary HAECs. (**a**) Representative fluorescence images of primary HAECs incubated with pHrodo Red dextran under control conditions (PBS) and following PHOH-001 treatment. pHrodo Red dextran fluorescence increases with decreasing pH and was used to assess endosomal acidification. PHOH-001 treatment resulted in reduced vesicular fluorescence intensity, consistent with reduced endosomal acidification. Nuclei were counterstained with DAPI. Merged images and corresponding magnified regions (zoom) are shown to highlight vesicular localization. Images were acquired at 60× magnification using an EVOS fluorescence microscope. Scale bars: 40 µm (merge) and 5 µm (zoom). (**b**) Quantification of pHrodo Red dextran fluorescence expressed as integrated density demonstrates reduced fluorescence in PHOH-001-treated cells relative to control. Data points are colour-coded by biological replicate. Data represent mean±sd from two biological replicates, each with ten technical replicates (*P*<0.05, two-tailed unpaired Student’s t-test).

To evaluate whether PHOH-001 modulates endosomal pH, primary HAECs were treated with PHOH-001 or control conditions and incubated with the pH-sensitive probe pHrodo Red dextran, in which vesicular fluorescence increases as pH decreases. Fluorescence microscopy revealed reduced pHrodo Red dextran signal in PHOH-001-treated cells compared to controls, consistent with decreased endosomal acidification (Fig. 5a). Magnified views of the same fields confirmed that this effect was observed across individual cells, suggesting a broader alteration in vesicular pH dynamics rather than localized changes. Quantification using Fiji software demonstrated a significant reduction in pHrodo Red dextran fluorescence intensity in PHOH-001-treated HAECs compared to controls (Fig. 5b). Together, these findings indicate that PHOH-001 is associated with altered endosomal pH in primary HAECs.

### PHOH-001 impairs early virus–cell interactions, replication and syncytium formation in primary HAECs

**Fig. 6.**
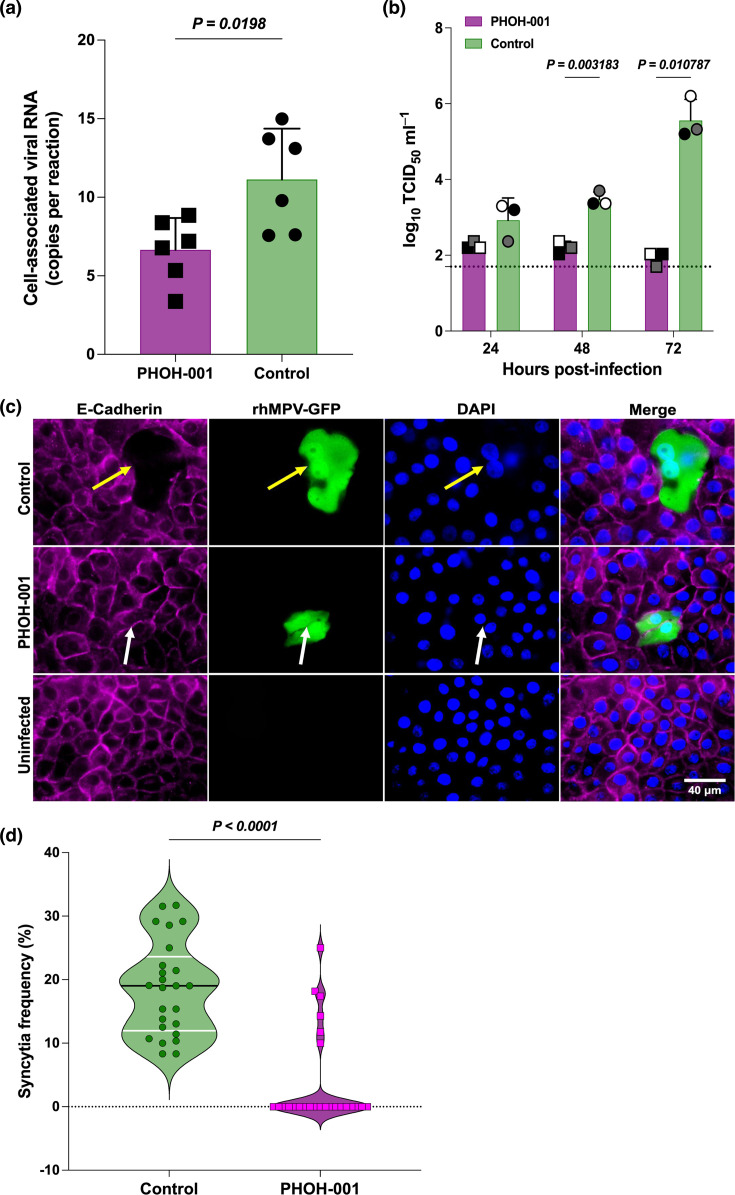
PHOH-001 modulates early virus–cell association and reduces hMPV replication and syncytium formation in HAECs. (**a**) Viral attachment assay performed at 4 °C to restrict infection to cell surface binding. HAECs were inoculated with rhMPV-GFP (m.o.i.=1) in the presence or absence of PHOH-001. Cell-associated viral RNA was quantified by qPCR. PHOH-001 treatment was associated with reduced viral RNA compared to control conditions. Data represent mean±sd from two independent experiments, each performed with one biological replicate and three technical replicates (*P*<0.05, two-tailed unpaired Student’s t-test). (**b**) TCID_50_ assay of hMPV replication in PHOH-001-treated HAECs. Infectious viral titres in supernatants from PHOH-001- and PBS-treated HAECs were measured at 24, 48 and 72 hpi. Data points are colour-coded by biological replicate. Data represent mean±sd from three biological replicates, each with four technical replicates. Statistical significance between PHOH-001 and PBS at each time point was determined using multiple unpaired two-tailed Student’s t-tests with Bonferroni correction for multiple comparisons (adjusted *P*<0.017) in GraphPad Prism. The dashed line indicates the LOD (1.7 log_10_ TCID_50_ ml⁻¹). (**c**) Representative fluorescence images of rhMPV-GFP-infected HAECs treated with or without PHOH-001 at 72 hpi. Multinucleated syncytia are indicated by yellow arrows, and individual infected cells are indicated by white arrows. Images were acquired at 60× magnification using an EVOS fluorescence microscope. Scale bar=40 µm. (**d**) Quantification of syncytium formation in HAECs. The percentage of DAPI-positive nuclei incorporated into syncytia was determined from fluorescence images using CellProfiler. Data are presented as violin plots, where individual points represent measurements; the black line indicates the median and white lines indicate the upper and lower quartiles. Data represent mean±sd from 25 technical replicates (*P*<0.05, two-tailed unpaired Student’s t-test).

To further define the antiviral effects of PHOH-001 on hMPV infection, we examined early virus–cell interactions, viral replication kinetics and syncytium formation. To assess early stages of infection, we performed a viral attachment assay under conditions that restrict internalization. PHOH-001 treatment was associated with reduced cell-associated viral RNA compared to controls, suggesting an effect on early virus–cell interactions (Fig. 6a). We next assessed viral titres using a TCID_50_ assay, which demonstrated a significant reduction in infectious viral titres in the supernatants of PHOH-001-treated HAECs compared to controls (Fig. 6b). This reduction in viral titres is consistent with reduced productive viral replication. Next, we examined syncytium formation, a hallmark of hMPV infection that is critical for viral dissemination within the host [17]. Syncytia are multinucleated giant cells formed when the virus induces fusion between infected cells and adjacent uninfected cells [15,40]. Fluorescence microscopy images showed that syncytia were notably reduced in PHOH-001-treated HAECs compared to PBS-treated controls, consistent with reduced viral spread (Fig. 6c). Quantification of syncytium formation in PHOH-001-treated and control cells revealed a significant reduction in syncytia in the treated samples, indicating that PHOH-001 is associated with altered viral fusion and propagation (Fig. 6d). These findings likely reflect downstream effects of earlier steps in infection. Taken together, these findings indicate that PHOH-001 does not completely inhibit hMPV infection but is associated with effects on both early virus–cell interactions and downstream stages of infection, as reflected by reduced viral replication and syncytium formation.

### PHOH-001 alters hMPV F protein localization and is associated with reduced viral spread

**Fig. 7.**
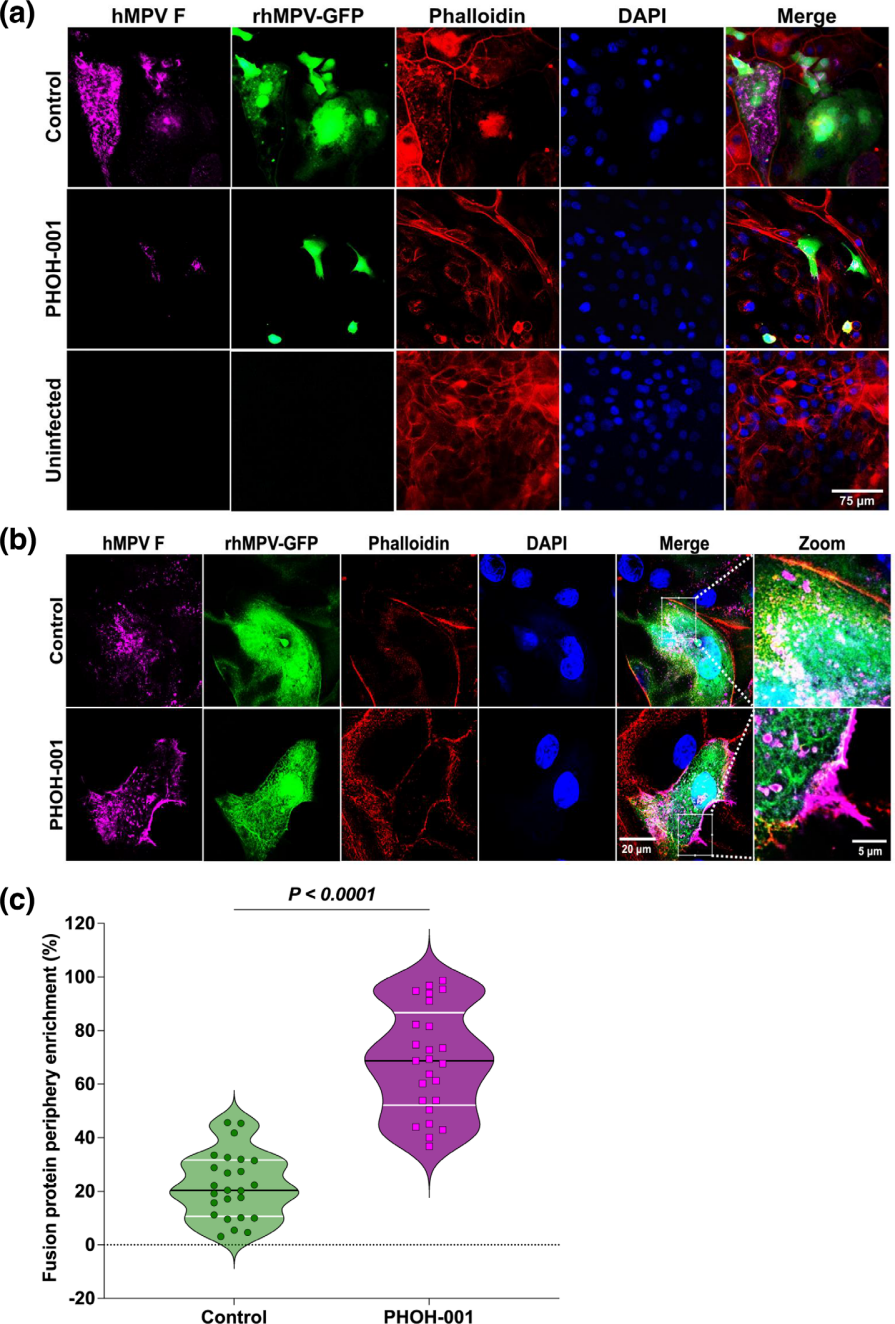
PHOH-001 alters hMPV F protein localization. (**a**) Representative low-magnification (20×) images of primary HAECs infected with rhMPV-GFP (m.o.i.=1) and treated with PHOH-001 or PBS at 72 hpi. Actin is visualized by phalloidin staining to delineate cellular morphology and was not independently quantified. PHOH-001 treatment was associated with altered actin organization compared to PBS controls. Scale bar=75 µm. (**b**) Representative high-resolution confocal images (63×) showing hMPV F protein distribution in infected HAECs at 72 hpi. In PBS-treated cells, F protein is broadly distributed throughout the cytoplasm and syncytia, whereas PHOH-001-treated cells exhibit pronounced enrichment of F protein at the cell periphery, proximal to the plasma membrane. Scale bar: 20 µm (merge) and 5 µm (zoom). (**c**) Quantification of F protein peripheral enrichment within GFP-positive infected cells using CellProfiler. Individual cells were segmented based on phalloidin staining, and integrated F protein fluorescence intensity was measured within whole-cell and plasma membrane-proximal peripheral regions. Peripheral enrichment was calculated as the ratio of peripheral to whole-cell integrated intensity on a per-cell basis. PHOH-001 treatment resulted in a significant increase in F protein peripheral enrichment compared to PBS-treated controls. Data represent individual data points with mean±sd from 25 technical replicates per condition (*P*<0.05, two-tailed unpaired Student’s t-test).

The hMPV F protein is essential for viral entry and cell-to-cell spread. To assess whether PHOH-001 affects F protein trafficking, primary HAECs were infected with rhMPV-GFP (m.o.i.=1) and treated with PHOH-001 or PBS. Low-magnification (20×) imaging revealed differences in actin organization between treatment groups, with PHOH-001-treated cells exhibiting altered cortical actin distribution compared to PBS controls (Fig. 7a), consistent with prior reports of actin reorganization during hMPV infection and studies demonstrating that actin dynamics influence pneumovirus replication and cellular architecture [41,42]. High-resolution confocal imaging at 72 hpi revealed marked differences in F protein distribution (Fig. 7b). In PBS-treated cells, F protein was broadly distributed throughout the cytoplasm, consistent with normal intracellular trafficking and syncytium formation. In contrast, PHOH-001-treated cells exhibited pronounced enrichment of F protein at the cell periphery, proximal to the plasma membrane. This redistribution coincided with changes in cortical actin organization. Quantitative image analysis was performed using CellProfiler to assess F protein localization within GFP-positive infected cells. Phalloidin staining was used to delineate individual cell boundaries and define whole-cell and plasma membrane-proximal peripheral regions of interest, minimizing over-segmentation of multinucleated syncytia. Integrated F protein fluorescence intensity was measured on a per-cell basis, and peripheral enrichment was calculated as the ratio of peripheral to whole-cell intensity. PHOH-001 treatment significantly increased peripheral F protein enrichment relative to PBS controls (Fig. 7c), consistent with altered F protein trafficking and/or retention at the plasma membrane. Consistent with these localization changes, PHOH-001 reduced F protein expression at both the transcript and protein levels, as measured by RT-qPCR and Western blot analysis (Fig. S3). These findings should be interpreted in the context of earlier stages of infection, as PHOH-001 was also associated with reduced virus–cell interactions and altered endosomal pH (Figs5  6), suggesting that the observed changes in F protein localization may reflect downstream consequences of effects on viral entry or intracellular processing. Collectively, these findings suggest that PHOH-001 resulted in altered F protein localization and actin organization, consistent with impaired syncytium formation and reduced viral spread.

## Discussion

hMPV is a significant respiratory pathogen, particularly in immunocompromised individuals, the elderly and young children, and there are currently no approved targeted therapeutics. Here, we show that PHOH-001, an inhaled alkaline buffer, reduces hMPV infection in primary HAECs under both submerged and ALI culture conditions, supporting modulation of airway epithelial pH as a potential antiviral strategy.

**Fig. 8.**
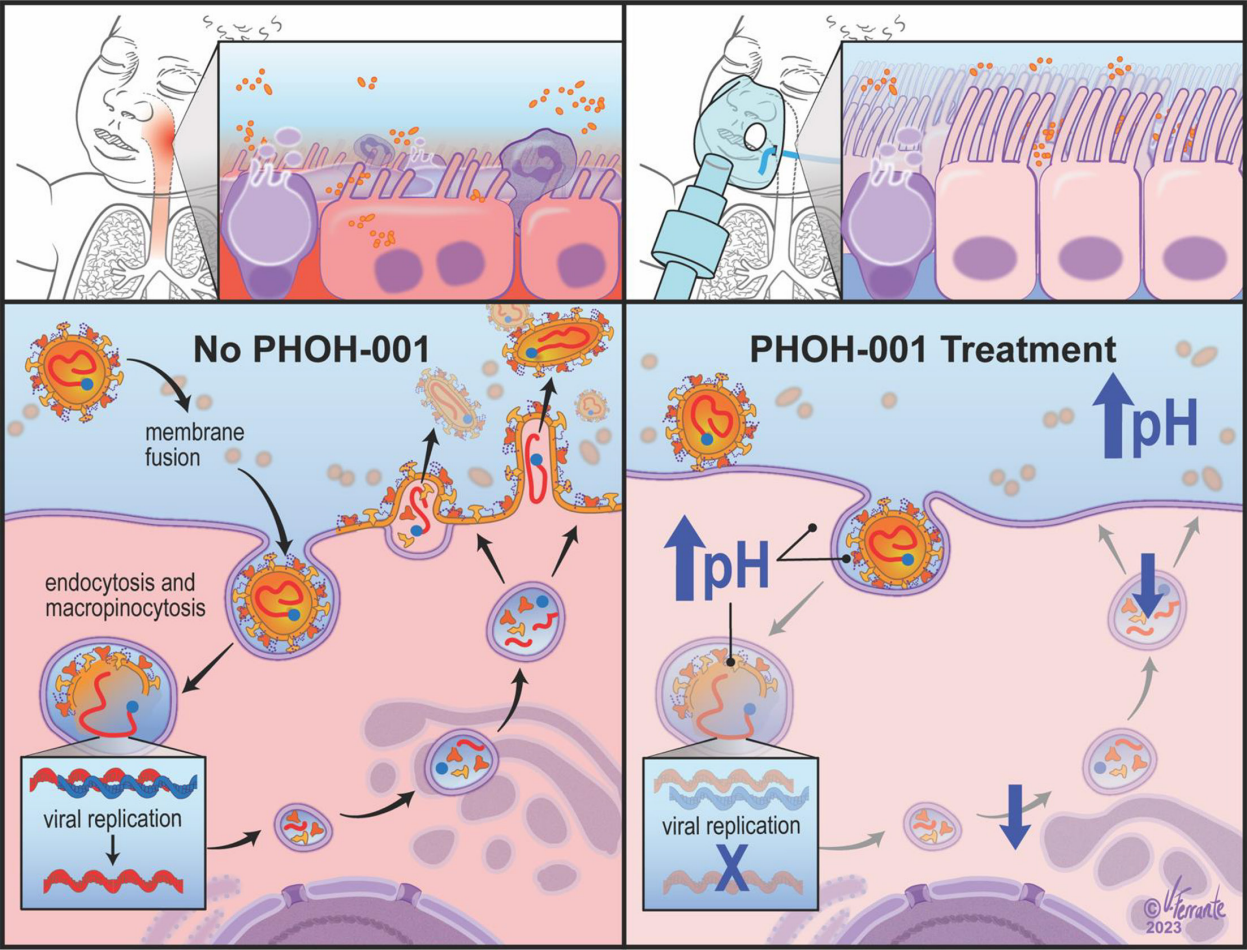
Proposed mechanism of PHOH-001’s action in modulating hMPV infection. Schematic representation of the hypothesized mechanism by which PHOH-001 modulates airway epithelial pH to inhibit hMPV infection. In the absence of PHOH-001 (left), hMPV can enter airway epithelial cells via endocytosis, where low pH in the endosome triggers conformational changes in the viral F protein, promoting membrane fusion and viral genome release. Subsequent F protein trafficking to the plasma membrane, a process supported by the host actin cytoskeleton, facilitates syncytium formation and viral egress, enabling efficient viral spread. In the presence of PHOH-001 (right), elevated pH is associated with reduced endosomal acidification, potentially reducing F protein activation and viral fusion. In addition, altered actin organization may further disrupt F protein trafficking and membrane dynamics, thereby impairing viral replication, syncytium formation and egress, ultimately restricting viral spread and infection.

Our findings support a mechanistic model in which PHOH-001 is associated with effects on multiple pH-dependent stages of the hMPV infection cycle (Fig. 8). In untreated cells, hMPV can enter via endocytosis, where acidic endosomal pH promotes conformational activation of the viral F protein, enabling membrane fusion, genome release and viral replication. Subsequent actin-dependent trafficking of F protein to the plasma membrane facilitates syncytium formation and viral egress, promoting efficient cell-to-cell spread. In PHOH-001-treated cells, elevated extracellular and intracellular pH was associated with reduced endosomal acidification and virus–cell interactions, consistent with effects on early stages of infection. Confocal imaging revealed accumulation of F protein at the cell periphery, with reduced cytoplasmic dispersion and limited syncytium formation, coinciding with markedly reduced infectious viral titres. Confocal imaging further demonstrated alterations in the cortical actin network following PHOH-001 treatment, consistent with prior reports that hMPV remodels actin to support intracellular trafficking and replication [41,42]. High-resolution imaging showed that although F protein localized proximal to the plasma membrane in PHOH-001-treated cells, it failed to achieve the broad membrane distribution characteristic of syncytium-competent infection. Importantly, these later phenotypes should be interpreted in the context of earlier stages of infection, as changes in F protein localization, actin organization and syncytium formation may reflect downstream consequences of viral entry or intracellular processing. Together, these data indicate that PHOH-001 is associated with altered F protein localization and actin organization, consistent with impaired viral replication, syncytium formation and egress.

PHOH-001 has been evaluated *in vivo* and shown a favourable safety profile in both healthy and asthmatic individuals, with no reported adverse effects [33,34]. Consistent with these findings, prior studies in primary HAECs demonstrated that PHOH-001 does not induce cytotoxicity or disrupt epithelial integrity at concentrations used in antiviral experiments [33], supporting the interpretation that reduced hMPV infection observed here reflects antiviral activity rather than nonspecific cellular toxicity. Cytotoxicity was not a primary endpoint of the present study; however, treated HAECs retained normal morphology throughout the experimental period, and no increase in LDH release was observed following PHOH-001 treatment across concentrations and time points (Fig. S4). Although *in vivo* efficacy against hMPV has not yet been demonstrated, the results from human safety trials support translational potential [33,34]. Furthermore, preliminary studies exploring PHOH-001 activity against other respiratory viruses, including SARS-CoV-2 and RSV, suggest that modulation of endosomal and airway pH may be a broadly applicable antiviral strategy [33,35]. Unlike direct antiviral inhibitors targeting viral proteins, modulation of airway pH represents a host-directed antiviral strategy that may reduce the likelihood of resistance development.

Limitations of this study include the use of a single hMPV A2 strain and exclusively *in vitro* HAEC models. Because pH sensitivity of hMPV fusion is strain dependent, evaluation across additional genotypes will be important to determine the breadth of this antiviral strategy. In addition, while reductions in syncytium formation and infectious viral titres suggest that PHOH-001 affects both early and post-entry stages of infection, the relative contribution of entry inhibition versus post-entry effects cannot be definitively resolved in the current study. Future studies incorporating time-of-addition approaches or direct measurements of entry and intracellular trafficking dynamics will be required to further define the stage(s) of inhibition.

In summary, PHOH-001 represents a novel approach to modulate airway epithelial pH and reduce hMPV infection through effects on multiple stages of the viral life cycle, including early virus–cell interactions, endosomal pH and downstream trafficking-associated processes. Our findings provide a mechanistic foundation and strong rationale for advancing PHOH-001 toward preclinical and clinical evaluation as a potential therapeutic for hMPV and other pH-dependent respiratory viral infections.

## Supplementary material

10.1099/jgv.0.002274 Supplementary Material 1.

## Abbreviations

ALI: air–liquid interface
ASL: airway surface liquid
DMEM: Dulbecco’s Modified Eagle Medium
HAEC: human airway epithelial cell
hMPV: human metapneumovirus
hpi: h post-infection
LDH: lactate dehydrogenase
LOD: limit of detection
PFA: paraformaldehyde
rhMPV: recombinant human metapneumovirus
RLU: relative light units
RSV: respiratory syncytial virus
RT-qPCR: reverse transcription quantitative PCR
SARS-CoV-2: severe acute respiratory syndrome coronavirus 2
