# Supplementary material for: A novel antiviral strategy targeting human metapneumovirus through pH modulation in human airway epithelial cells
Source: J Gen Virol. 2026 Jun 3;107(6):002274. doi: 10.1099/jgv.0.002274 (PMC13232866; doi:10.1099/jgv.0.002274)

Supplemental Materials

Table S1. Donor demographics for primary HAECs including age, sex, disease state, and origin.

Donor #	Sex, Age	Disease State	Origin
#07783	Male, 12 years	Healthy	Bronchial
#0059	Female, 25 years	Healthy	Bronchial
#0098	Male, 42 years	Healthy	Bronchial

Table S2. Primer, probe, and gene block design for CAN97-83 [A2] fusion protein RT-qPCR.

**Name:**	**Sequence (5'-3')**
Forward Primer (fusion protein)	AATTGCCAAAACCATCCGGC
Reverse Primer (fusion protein)	CACTGCAGTTGCCAACACTC
Probe (fusion protein)	6FAM-TCACAGCAATTAAGAATGCCCTCAAACGACCAATGAA-MGBNFQ
Gene Block (fusion protein)	AATAGCACTCGGTGTTGCAACAGCAGCTGCAGTCACAGCAGGTGTTGCAATTGCCAAAACCATCCGGCTTGAGAGTGAAGTCACAGCAATTAAGAATGCCCTCAAAACGACCAATGAAGCAGTATCTACATTGGGGAATGGAGTTCGAGTGTTGGCAACTGCAGTGAGAGAGCTGAAAGACTTTGTGAGC

Figure S1. Validation of rhMPV-GFP replication kinetics in Vero E6 cells. (A) Representative fluorescence images of Vero E6 cells infected with rhMPV-GFP at a MOI of 0.01, 0.1, or 1. Images were captured at 18, 24, 48, and 72 hpi at 2x magnification. Scale bar = 1250 µm. **(B)** Dual-axis quantification of GFP signal (RLU; left y-axis) and infectious titers ($TCID_{50}$ mL^{-1}; right y-axis) at 18, 24, 48, and 72 hpi. GFP signal was measured directly from the assay plate using a plate reader, and infectious titers were determined from supernatant collections by $TCID_{50}$ assays on Vero E6 cells. Data represent mean ± SD from three technical replicates. RLU and $TCID_{50}$ increased in a MOI- and time-dependent manner, validating assay performance and supporting subsequent application in primary HAECs. The dashed line indicates the LOD of the $TCID_{50}$ assay (1.7 log_{10} $TCID_{50}$ mL^{-1}). Values below the limit of detection were plotted at the LOD.

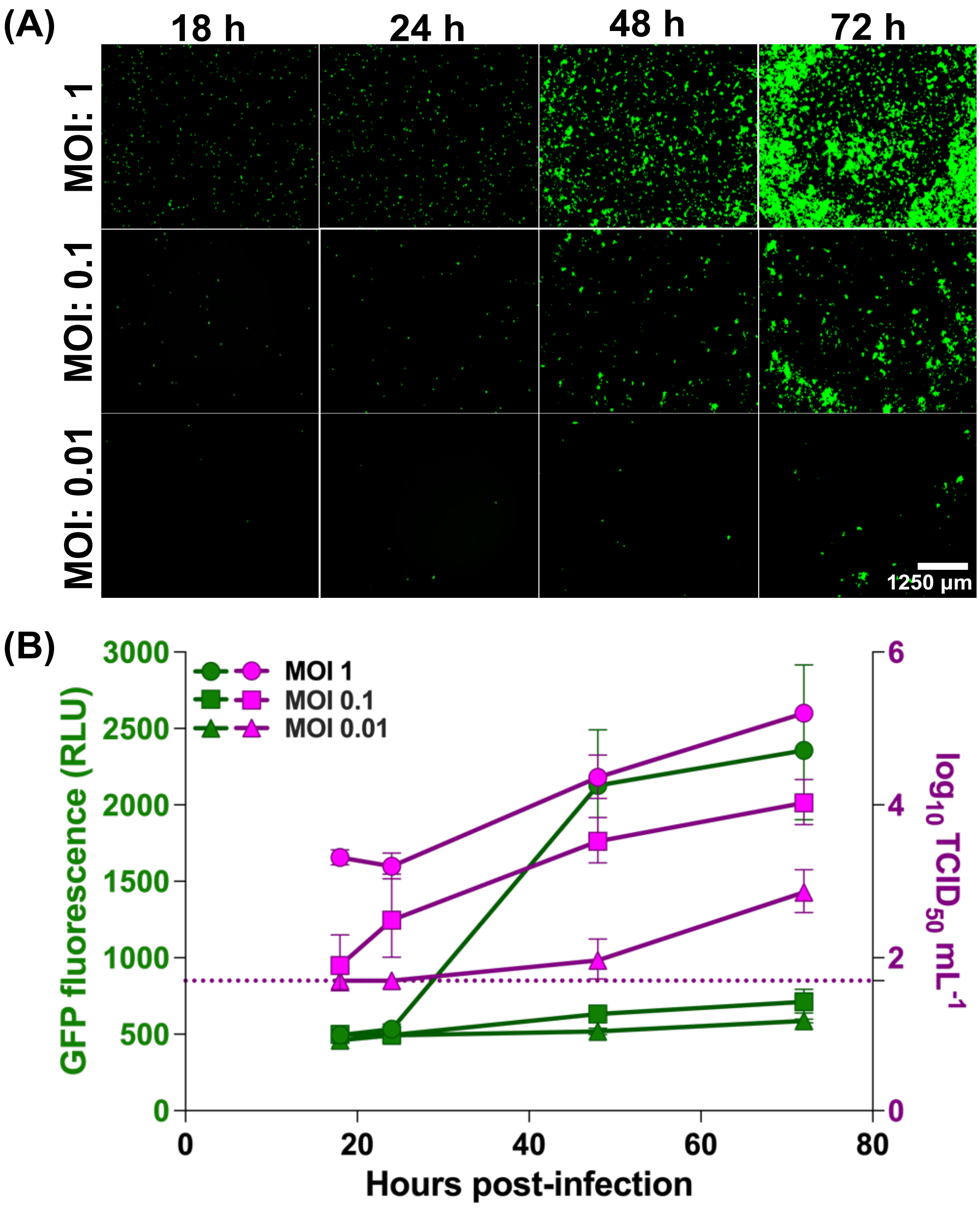

Figure S2. Western blot and RT-qPCR validation of dose-dependent hMPV inhibition by PHOH-001. (A) Western blot analysis of primary HAECs infected with rhMPV-GFP (MOI = 1) and treated with increasing concentrations of PHOH-001. A narrower dose range was used to validate the fluorescence-based inhibition observed in Figure 4. Blots show reduced GFP expression with increasing PHOH-001 concentration. **(B)** Quantification of GFP expression normalized to β-actin confirms a significant, dose-dependent reduction in GFP levels with PHOH-001 treatment. Data represent four technical replicates and were analyzed using one-way ANOVA with post hoc Tukey's test ($p < 0.05$). **(C)** RT-qPCR quantification of the hMPV F protein gene revealed a dose-dependent reduction in hMPV F gene expression following PHOH-001 treatment. As PHOH-001 concentration increased, F protein gene expression progressively decreased, consistent with inhibition of hMPV replication at higher PHOH-001 doses. RT-qPCR data represent three technical replicates and were analyzed using one-way ANOVA with post hoc Tukey's test ($p < 0.05$).

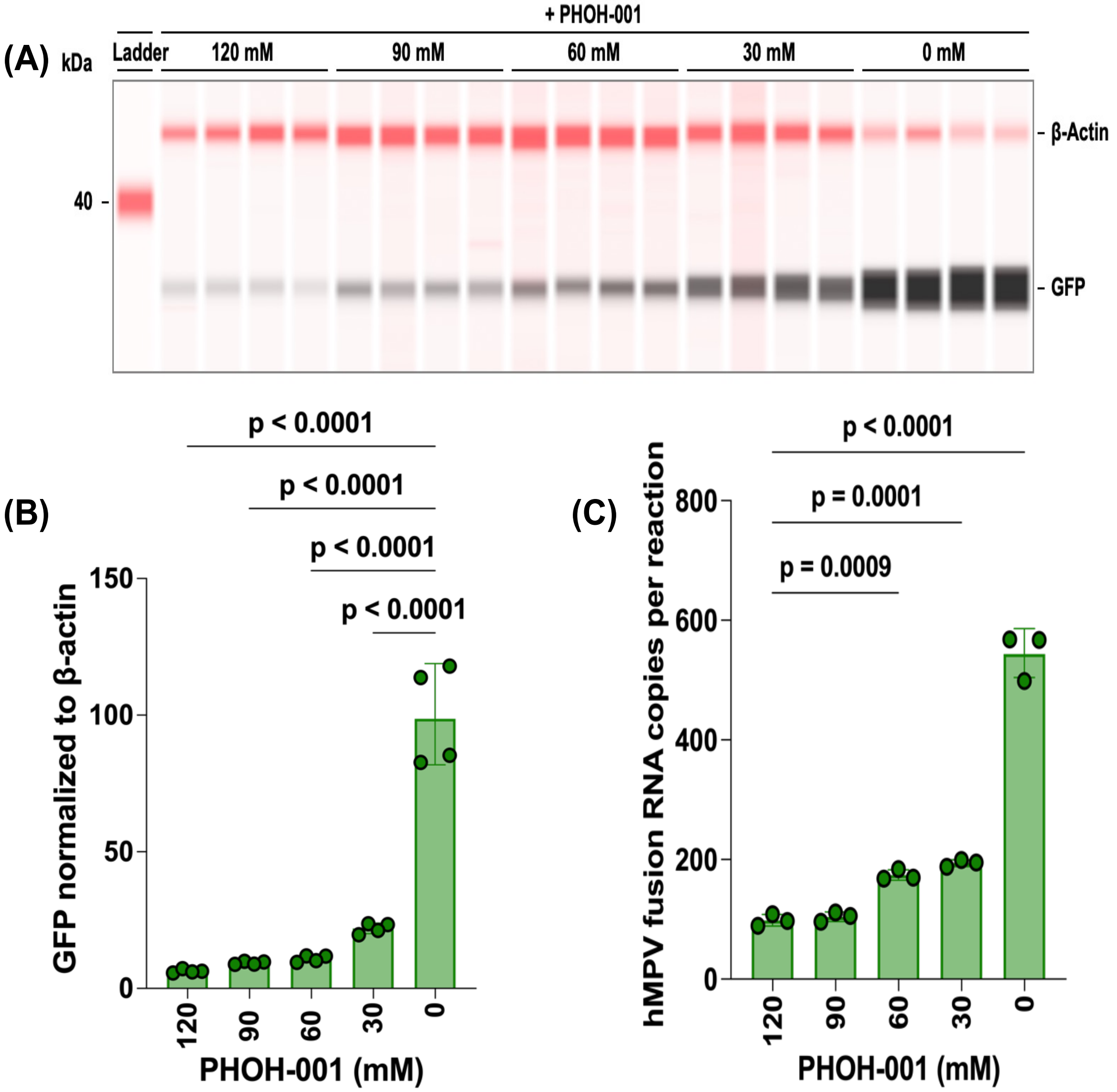

Figure S3. Western blot and RT-qPCR confirmation of reduced F gene and protein expression in HAECs treated with PHOH-001. (A) Western blot analysis of primary HAECs infected with rhMPV-GFP (MOI = 1) comparing PHOH-001-treated and PBS-treated groups. Blots show reduced hMPV F protein levels in PHOH-001-treated samples relative to controls. **(B)** Quantification of hMPV F protein expression normalized to β-actin confirms a significant reduction in PHOH-001-treated samples. Data represent three technical replicates and were analyzed using one-way ANOVA with post hoc Tukey's test ($p < 0.05$). **(C)** RT-qPCR analysis of hMPV F gene expression in PHOH-001-treated versus untreated HAECs. Results show a significant decrease in hMPV F gene transcript levels in the treatment group, consistent with the protein-level reduction observed in panel A. Data represent six technical replicates and were analyzed using one-way ANOVA with post hoc Tukey's test ($p < 0.05$).

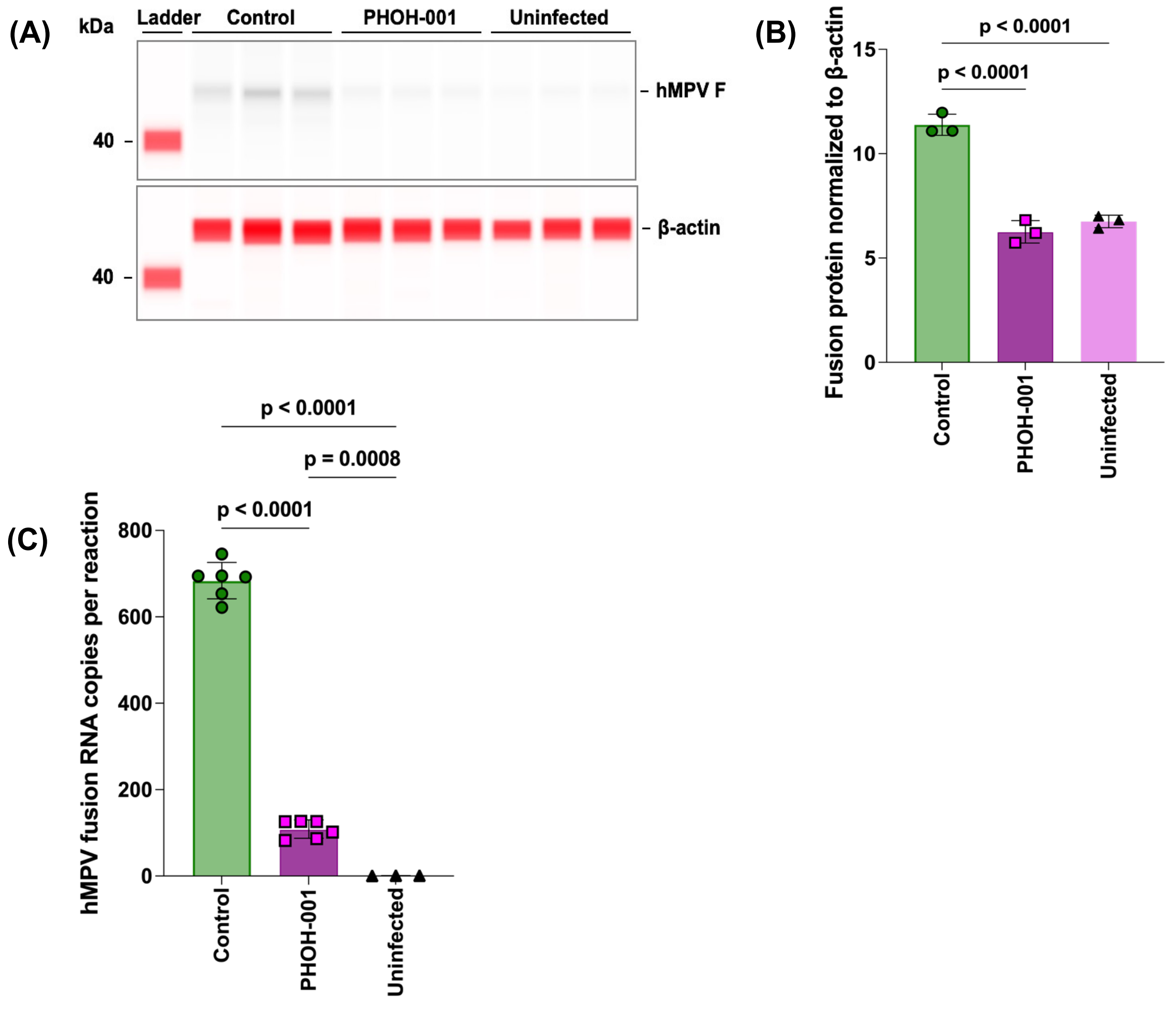

Figure S4. PHOH-001 does not induce cytotoxicity in primary HAECs. LDH release was measured at 4, 12, and 24 hours following treatment with increasing concentrations of PHOH-001. Cytotoxicity was calculated relative to spontaneous and maximum LDH release controls. No consistent or dose-dependent increase in LDH release was observed across concentrations or time points. Variability observed in untreated (0 mM) controls at select time points likely reflects baseline fluctuations in spontaneous LDH release rather than PHOH-001-associated cytotoxicity. Values represent percent cytotoxicity calculated from the mean of three technical replicates per condition. Negative values were set to zero for visualization.

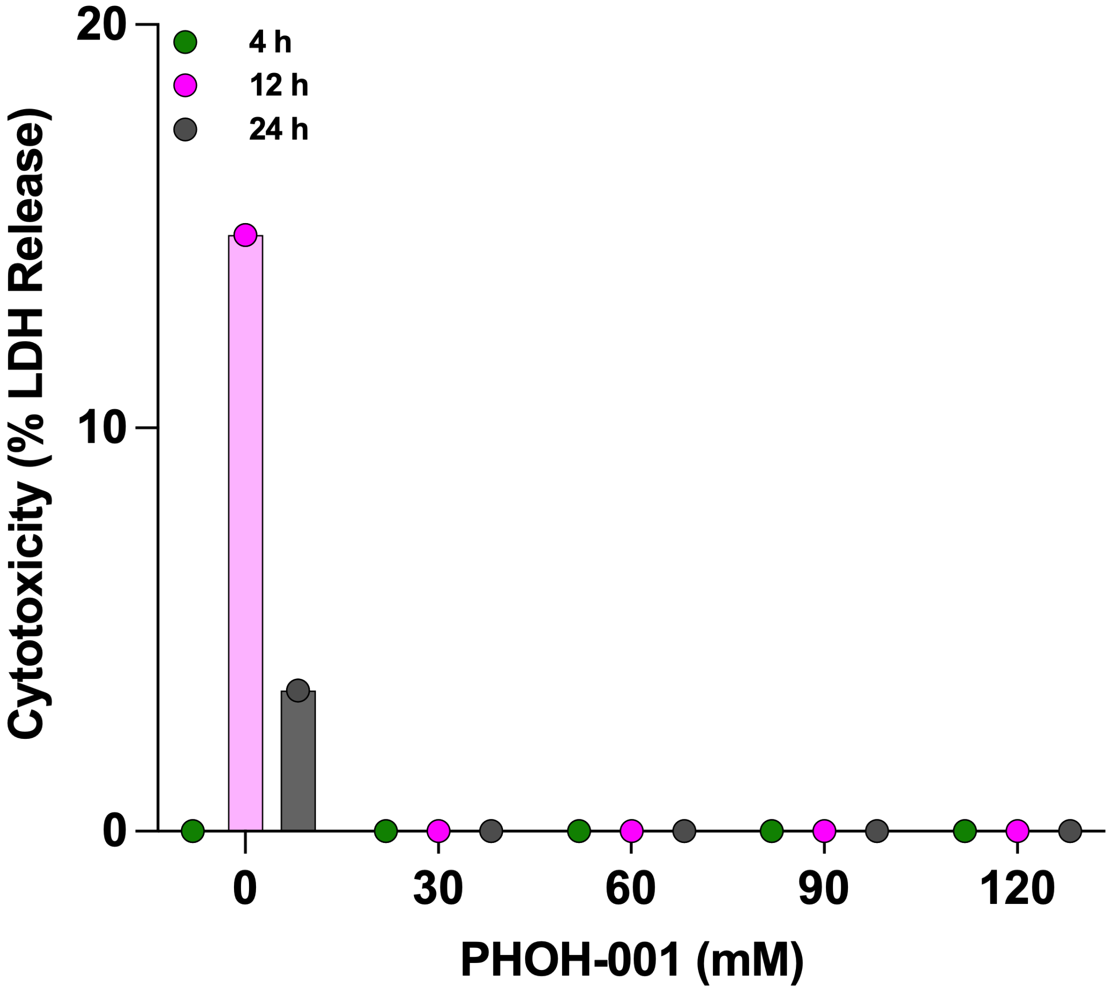

Supplement: Supplementary Material 1. [file jgv-107-02274-s001.pdf]
